# UK recommendations for the management of transgender and gender-diverse patients with inherited cancer risks

**DOI:** 10.1038/s44276-023-00002-0

**Published:** 2023-06-22

**Authors:** Josephine Giblin, Beth Coad, Catherine Lamb, Cheryl Berlin, Gillian Rea, Helen Hanson, Katie Snape, Alison Berner, Consensus Meeting attendees

**Affiliations:** 1 Central & South Genomic Medicine Service Alliance, Southampton, UK; 2 South West Thames Regional Genomics Service, St George’s Hospital NHS Foundation Trust, London, UK; 3 Nottingham Clinical Genetics Service, Nottingham University Hospitals NHS Trust, Nottingham, UK; 4 North West Thames Regional Genetics Service, London North West University Healthcare NHS Trust, London, UK; 5 Northern Ireland Regional Genetics Service, Belfast Health and Social Care Trust, Belfast, UK; 6 Barts Cancer Institute, Queen Mary University of London, London, UK

## Abstract

As the rate of people openly identifying as transgender or gender diverse (TGD) is increasing, UK cancer genetics services are seeing growing numbers of TGD patients. Lack of appropriate clinical guidelines and a scarcity of robust data about the impact of gender-affirming treatments on cancer risk has led to uncertainty of how best to support TGD patients, and inequity in standards of care. To address this gap, the UK Cancer Genetics Group and Central & South Genomic Medicine Service Alliance facilitated a 2-day meeting to develop national consensus to support the management of TGD patients with inherited cancer risks. Key stakeholders from a broad range of clinical specialties, patients advocates, and those with lived experience discussed and voted on recommendations for best practice. The consensus was reached on topics including family history questionnaires, pedigrees, clinical information, breast tissue management, gynaecological and prostate management, patient pathways, and education. Further work is required to reach consensus on the breast screening recommendations for TGD patients assigned female at birth who have had masculinising chest surgery. Here we present a summary of the processes used to reach consensus, and the recommendations from this meeting.

## Introduction

**Fig. 1:**
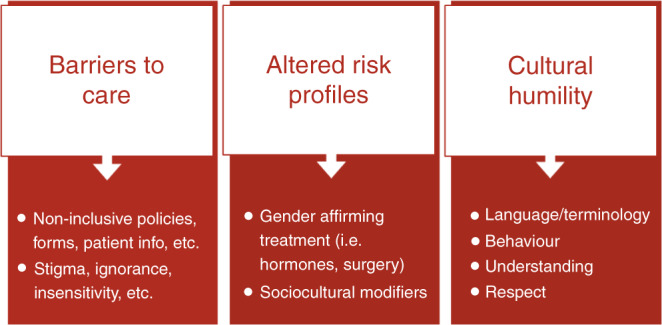
Factors to consider. Including barriers to care faced by TGD individuals, potentially altered risk profiles, and cultural humility of healthcare professionals.

Across the UK, genetics services have reported increasing numbers of transgender and gender-diverse (TGD) patients referred for advice regarding their inherited cancer risks. While a patient’s gender identity and gender expression may not alter the clinical approach in many scenarios, within cancer genetics clinics in particular, the needs of TGD patients may differ from cisgender patients [1]. Genetic clinicians should work to ensure they are providing an accessible service, equitable care, accurate risk assessments and appropriate recommendations regardless of gender identity and expression. These factors are complex and interconnecting and include those summarised in Fig. 1. For a glossary of terms used in this article, please see Table 1.

**Table 1:** Glossary of terms.

Term	Definition
Cisgender (or cis)	Someone whose gender identity is the same as the sex they were assigned at birth
Gender-affirming treatment (GAT)	Medical interventions that aim to align a person’s characteristics with their gender identity, such as hormone treatment or surgeries
Gender diverse	Usually used to describe a population, but can also refer to someone whose gender identity or gender expression does not conform to socially defined binary gender norms
Gender dysphoria	Psychological distress or discomfort experienced because of a mismatch between sex assigned at birth and gender identity
Gender expression	How a person chooses to outwardly express their gender, within the context of societal expectations of gender
Gender history	Information related to a TGD person’s past social, legal and/or medical transition
Gender identity	A person’s innate sense of their own gender, which may or may not correspond to the sex assigned at birth
LGBTQ +	Initialism for lesbian, gay, bisexual, transgender, queer and a wide range of other sexual and gender minorities
Non-binary	An umbrella term for people whose gender identity does not sit comfortably with binary “male” or “female”
Pronoun	Words used to refer to people’s gender in conversation (i.e., he, she, they)
Sex assigned at birth	The label a medical professional gives to a baby when it is born (i.e., male, female, intersex), usually based on external genitalia. AFAB and AMAB are commonly used acronyms meaning “assigned female at birth” and “assigned male at birth”, respectively.
Transgender man	A term used to describe someone who is assigned female at birth but identifies and lives as a man. May be shortened to trans man
Trans/TGD status	Description of someone’s status as transgender or gender diverse
Transgender woman	A term used to describe someone who is assigned male at birth but identifies and lives as a woman. May be shortened to trans woman
Transfeminine	Describes someone who was assigned male at birth but identifies more with a feminine identity
Transgender (or trans)	An umbrella term to describe people whose gender is not the same as, or does not sit comfortably with, the sex they were assigned at birth
Transitioning	The steps a trans person may take to live in the gender with which they identify
Transmasculine	Describes someone who was assigned female at birth, but identifies more with a masculine identity
Transphobia	Discrimination against trans people

Clinicians have reported uncertainty about how to best support TGD patients, with cases requiring extensive review of limited literature, multiple ad hoc discussions with specialist clinicians, and case presentation at National UK Cancer Genetics Group (UKCGG) multidisciplinary team (MDT) meetings. These cases have been highlighted:

- A lack of appropriate guidelines.
- Inadequate patient care pathways due to limited clear communication between genetics and gender identity services, exacerbated by the long wait times for appointments with each service.
- Uncertainty about how to record TGD patients in pedigrees.
- Insufficient understanding of where clinical genetics advice might influence gender-affirming care (and vice versa).
- Non-inclusive policies, forms, patient information leaflets, etc.
- Minimal education for clinicians regarding TGD-specific healthcare needs and cultural competency.

To address this, a national consensus meeting was held on the mornings of the 6th and 7th of October 2022, with support from the UKCGG and Central & South Genomic Medicine Service Alliance (CAS GMSA). This consensus meeting brought together key stakeholders to address the above challenges, with the aim of:

- Establishing consensus guidelines for the clinical management of TGD adults with inherited cancer risk.
- Providing an educational opportunity for healthcare professionals.
- Beginning a sustainable collaboration between cancer genetics and gender identity services, and their service users.
- Forming a framework for continuous improvement.

## Methods

### Development of proposed statements for best practice

A preliminary service evaluation was carried out to examine whether three UK Cancer Genetics Services were meeting the needs of TGD patients referred with a personal and/or family history of cancer. The results were collated and discussed by a specialist “consensus working group” (JG, AB, CB, CL, GR) to identify areas for service improvement and where formal guidance was lacking. Proposed statements for best practice were developed by the consensus working group and sent to HH and KS as for review, as representatives of the UKCGG. Proposed statements spanned seven topics: Family History Questionnaires, Pedigree Charting, Clinical Information, Breast Tissue Management, Gynaecological and Prostate Management, Patient Pathways and Education.

### Pre-meeting preparation

Invitations to register to attend the consensus meeting were sent to key stakeholders and clinicians with specialist expertise. Background documents (available at https://www.ukcgg.org/information-education/ukcgg-consensus-meetings/) were produced and circulated to all registrants prior to the meeting. These documents were designed to provide attendees (who had a variety of backgrounds) with a minimum understanding of inherited cancer predisposition, TGD-specific healthcare issues, and how these two subjects intersect in the context of the specific topics covered in the meeting.

### Format

The meeting was held virtually via Zoom over two consecutive mornings and moderated by the working group. The agenda (see supplementary) for the meeting was divided into seven sessions in line with the topics of the proposed statements. Each session started with a talk from an expert speaker, followed by group discussion, and finally voting on each proposed statement for best practice.

### Presentation of current knowledge

The talk for each topic was provided by a speaker with expertise in the subject area. The aim of each talk was to review the issues to be discussed and the available evidence, equipping attendees with information with which to base their votes on each proposed statement. Time was allowed for attendees to ask questions to speakers.

### Group discussions

Following each talk, attendees were presented with the proposed statements for the topic and divided into five small “breakout groups” to discuss the statements. Each group was moderated by a member of the consensus working group, who made notes of the discussion. After this, the full meeting was reconvened, and a representative of each breakout group relayed the key points of their discussion to all attendees for further discourse. Where indicated, wording of the proposed statement was adapted based on the group discussion.

### Voting

Voting on each proposed statement was carried out using Slido [2], which allows real-time voting online. Most statements were presented with five options for voting using a Likert scale of “strongly agree”, “agree”, “neither agree nor disagree”, “disagree” and “strongly disagree”. Proposed statements were considered to reach consensus if 80% of respondents voted “strongly agree” or “agree”, when a minimum of 80% of attendees (at the time of voting) had cast their vote. If consensus was not reached on a statement, attendees further discussed the statement and phrasing was altered in real-time until consensus was reached (if consensus could be reached within the time constraints of the meeting). Two statements did not use the Likert scale, and instead gauged attendees’ preferences from a small number of options.

### Meeting report

Following the meeting, a summary of the agreed statements of best practice were circulated to attendees, posted on the UKCGG website, and presented at the UKCGG Winter Meeting 2022 for further comments to ensure that statements were an accurate representation of the consensus reached.

## Results and discussion

### Attendees

Eighty-nine stakeholders attended from across the UK, Ireland, and Amsterdam. Sixteen attendees were TGD (18%), and 73 were cisgender (82%). Attendees included genetic counsellors (*n* = 35), clinical geneticists (*n* = 20), gender identity specialists (*n* = 15), charity representatives (*n* = 6), patients and members of the public (*n* = 6), clinicians from breast screening and radiology (*n* = 2), surgeons (*n* = 2), representatives from NHSE (*n* = 2) and researchers (*n* = 1). Each of the 24 UK regional genetics services was represented, as well as the regional service for the Republic of Ireland. As the meeting focussed on guidelines for TGD adults, attendees did not include clinicians from The Gender Identity Development Service (GIDS), or young people with lived experience.

### Poll results

The consensus was reached on 32 of 33 proposed statements for best practice. Statements on which consensus was reached are summarised in Table 2. The consensus was not reached on one statement; “It is best practice (based on current evidence) for TGD patients who were assigned female at birth, and have had gender-affirming chest surgery, to be offered the same breast screening as cisgender men” (24% strongly agree; 46% agree; 22% neither agree nor disagree; 8% disagree; 0% strongly disagree; *n* = 67). Further comments on each topic follow below, using the same numbering and headings.

**Table 2:** Statements on which consensus was reached.

**1. Family history questionnaires**
It is best practice for family history questionnaires to include questions asking the probands’ gender identity and trans status, in line with the LGBT Foundation “Good practice guide to monitoring sexual orientation and trans status 2021” 72% strongly agree; 26% agree (98% consensus, *n* = 64) It is best practice for family history questionnaires to include a question asking the proband’s sex assigned at birth 76% strongly agree; 22% agree (98% consensus, *n* = 63) It is best practice for family history questionnaires to include an explanation as to why questions about trans status, gender identity, and sex assigned at birth are being asked 83% strongly agree; 17% agree (100% consensus, *n* = 59) It is best practice for family history questionnaires to include a free text box where the proband can include any other information about themselves or their family that they would like genetics to know, or that they feel would be relevant to their risk assessment 61% strongly agree; 37% agree (98% consensus, *n* = 65) It is best practice for family history questionnaires to include space for proband’s title and pronouns in personal details sections 80% strongly agree; 20% agree (100% consensus, *n* = 60)
**2. Pedigrees**
The shape that corresponds with the patients’ gender identity should be used when denoting a transgender man or transgender woman, with sex assigned at birth annotated beneath the symbol 47% strongly agree; 47% agree (94% consensus, *n* = 59) An additional symbol (other than the existing symbols such as square, circle, diamond, triangle etc.) should be available to denote non-binary and other gender-diverse identities, with sex assigned at birth annotated beneath the symbol 49% strongly agree; 38% agree (87% consensus, *n* = 65) Until further research and/or collaboration involving PPI and clinicians has been carried out, a hexagon should be used to denote non-binary and other gender-diverse identities, with sex assigned at birth annotated beneath the symbol 34% strongly agree; 50% agree (84% consensus, *n* = 62) Annotation used should be sex assigned at birth, not sex chromosomes, or other annotations such as “male to female” (“MTF”) or “female to male” (“FTM”) 67% strongly agree; 33% agree (100% consensus, *n* = 57) Patients should be advised why annotation of sex assigned at birth on pedigree may be clinically important information 80% strongly agree; 20% agree (100% consensus, *n* = 56) Where pedigree software does not allow for representing TGD patients as described above, clinicians should make efforts to manually correct the pedigree. If this is not possible or clinically safe, this should be recorded into the genetics notes 33% strongly agree; 57% agree (90% consensus, *n* = 61) Where pedigree software does not allow for representing TGD patients as described above, genetics services should encourage software developers to consider these features 65% strongly agree; 30% agree (95% consensus, *n* = 57)
**3. Clinical information**
Clinicians should use the title, name, pronouns, and family relationship terms that patients have stated their preferences for. If there is uncertainty over which terms the individual wishes to be used, this should be politely clarified 71% strongly agree; 27% agree (98% consensus, *n* = 55) It is best practice for current genetics records of name, gender, and title to be updated promptly on request of the patient, where possible. If this is not possible this should be documented 69% strongly agree; 25% agree (94% consensus, *n* = 55) Information about gender diversity should be received non-judgementally 83% strongly agree; 17% agree (100% consensus, *n* = 58*)* Clinicians should seek patient consent before recording, storing, or sharing information about gender history. Gender history should be treated with appropriate confidentiality as per national guidance 46% strongly agree; 38% agree (84% consensus, *n* = 63) Clinicians should ensure that individuals understand the purpose of clinical questions that are asked and examinations that are performed from the beginning of an appointment 67% strongly agree; 31% agree (98% consensus, *n* = 54) Clinicians should not unnecessarily ask personal information about patients’ gender history where this is not relevant to their care (for example, details about breast/chest surgery are not relevant to serrated polyposis syndrome) 43% strongly agree; 45% agree (88% consensus, *n* = 53) If potentially sensitive information about gender history is clinically relevant (e.g., in context of breast, prostate, and gynaecological cancer risk) questions should be asked clearly and directly, to avoid making assumptions 60% strongly agree; 38% agree (98% consensus, *n* = 50) Clinically relevant information includes (but is not limited to): • Past, current & planned gender-affirming hormones • Previous & planned gender-affirming surgeries • Gamete storage • Whether patient is under the care of a gender identity specialist (GIS), is on the waitlist for a GIS, or has been discharged back to GP from GIS 26% strongly agree; 67% agree (98% consensus, *n* = 54)
**4. Breast tissue management**
It is best practice (based on current evidence) for TGD patients who were assigned female at birth, and have remaining breast tissue (i.e., not had gender-affirming chest surgery), to be offered the same breast screening as cisgender women with equivalent risk (including population, moderate, high, and very high-risk screening) 52% strongly agree; 42% agree (94% consensus, *n* = 66) It is best practice (based on current evidence) for TGD patients who were assigned male at birth, and have developed additional breast tissue because of ≥5 years use of exogenous hormones, to be offered the same breast screening as cisgender women with equivalent risk (including population, moderate, high and very high-risk screening) 52% strongly agree; 45% agree (97% consensus, *n* = 71) TGD patients should be signposted to inclusive breast/chest awareness resources (where clinically appropriate) 85% strongly agree; 15% agree (100% consensus, *n* = 71) If risk-reducing mastectomy is in alignment with a patient’s plans for gender-affirming care, it may be appropriate to consider such surgery at a younger age than is often typical (on a case-by-case basis) 37% strongly agree; 51% agree (88% consensus, *n* = 71) It is best practice to use a shared decision-making model for breast/chest surgical management, involving the patient, gender identity specialists, surgeons, and clinical genetics 89% strongly agree; 11% agree (100% consensus, *n* = 72)
**5. Gynaecological and prostate management**
If risk-reducing tubal and/or ovarian surgery and/or hysterectomy is in alignment with a patient’s plans for gender-affirming care, it may be appropriate to consider such surgery at a younger age than is often typical (on a case-by-case basis) 39% strongly agree; 52% agree (91% consensus, *n* = 69) It is best practice for trans and gender-diverse patients with a prostate and a high-risk inherited predisposition to prostate cancer to have a discussion about prostate screening and be referred to a specialist clinic where possible 67% strongly agree; 32% agree (99% consensus, *n* = 69)
**6. Patient pathways**
Where applicable, clinicians relevant to patients’ gender care should be consulted by clinical genetics, with the consent of the patient 39% strongly agree; 50% agree (89% consensus, *n* = 66) Inherited cancer predisposition should not be a barrier to accessing gender-affirming treatment. Instead, clinicians should provide information about risks for patients to make informed decisions about their care, acknowledging if there is limited data currently available 56% strongly agree; 42% agree (98% consensus, *n* = 66) If genetic input is delaying gender-affirming treatment, clinical genetics should consider treating referrals with priority (on a case-by-case basis) 43% strongly agree; 49% agree (92% consensus, *n* = 61) If gender specialist input is delaying timely cancer risk reduction, clinical genetics should consider requesting priority review from gender identity services (on a case-by-case basis) 45% strongly agree; 49% agree (94% consensus, *n* = 65) A multidisciplinary team meeting involving relevant specialists (i.e., clinical genetics, gender identity specialists, surgeons, endocrinologists, radiologists) is desired in the form of… • A regularly scheduled national MDT meeting, comprised of centralised experts and clinicians relevant to the cases discussed 77% • Ad hoc MDT meetings as required, comprised of appropriate local specialists and clinicians relevant to the cases discussed 23% • Not desired 0% (*n* = 66) Any multidisciplinary team meeting involving relevant specialists should be adequately funded and resourced 90% strongly agree; 10% agree (100% consensus, *n* = 60)
**7. Education**
Relevant education is desired for: • Genetic Counsellors in training 84% • Clinical Geneticists in training 85% • Genetics Clinicians throughout their career in the form of continuing professional development 99% (*n* = 68)

### Family history questionnaires

Family history questionnaires (FHQs) can gather data about trans status, which is often lacking in medical record systems. Expanding FHQs to gather such data can aid risk assessment where TGD patients may not have felt comfortable disclosing this in person, and indicate a service is respectful of, and sensitive to, TGD people [1]. TGD and cis patients perceive questions about names, pronouns, and gender identity on clinical intake forms as acceptable and relevant to care, and reported greater comfort when information was collected in this manner [3, 4].

**Fig. 2:**
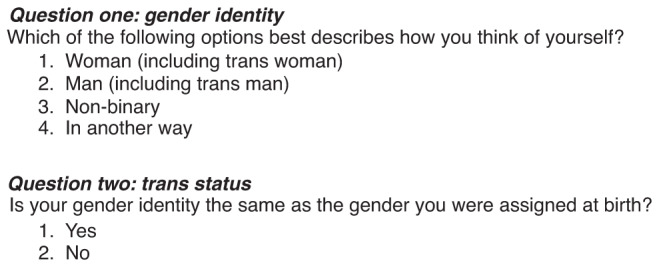
Two-step question for asking gender identity and trans, adapted from LGBT Foundation. Question one asks about gender identity, and question two asks trans status.

Cultural competence is required to ensure inclusivity and accurate data collection from FHQs. Information Standards help health and social care services to collect and process information in a consistent way. These are currently available for sexual orientation monitoring in the UK [5]. However, there is currently no national Information Standard for trans status monitoring. Whilst work is ongoing to develop a standardised approach, the LGBT Foundation have developed best practice guidelines with input from varied stakeholders [6]. The consensus was reached that FHQs should ask questions about gender identity and trans status using two-step questions, in line with LGBT Foundation guidance (Fig. 2).

This group also felt it was appropriate for FHQs to include a further question about sex assigned at birth, as clarity may be required for tissue-specific risk assessment. This group agreed an explanation is required for why gender identity, trans status, and sex assigned at birth were being asked (see example in supplementary). This would ensure that patients understand this information is being asked to inform care, and to clarify if it will be used in monitoring for audit or research. Furthermore, consensus was reached that it was appropriate for FHQs to include space for the proband to disclose their pronouns and title if they wish. This is a further signal of respect for TGD patients and is arguably relevant information for all patients. It was acknowledged that electronic healthcare records and pedigree software used by clinical genetics service may require development to effectively utilise this additional information collected in FHQs.

There was debate as to whether FHQs should gather trans status of all relatives, in addition to the proband. This may be directly relevant to cancer risk assessment. For example, a relative with breast cancer may have more bearing on the risk assessment if they were a trans woman assigned male at birth than if they were a cis woman. Although not unlawful for a proband to disclose a relatives trans status, it was agreed that it was unethical for this to be actively encouraged by clinicians, as an individuals’ decisions around disclosure are inherently personal, and there could be unforeseen consequences. Instead, this group considered it more appropriate to include adequate free text space for the proband to disclose any other details about themselves or their family, which they feel may benefit the risk assessment or genetic counselling process. This could be relatives’ trans status, their own gender-affirming treatment history, and other information not specific to gender history, such as adoption and family dynamics.

### Pedigree charting

A variety of symbols have been suggested for representing TGD patients (see Table 3). However, to date, there has been no UK consensus. This creates potential for confusion amongst clinicians, and potentially inaccurate cancer risk assessments and inappropriate recommendations. Clinical confusion and hesitation of how to represent TGD patients could also lead to the patient feeling invalidated, and less trusting of their clinician. TGD participants interviewed by Barnes et al. [7] highlighted the importance of validating their gender identity for creating a safe space in genetics clinics.

**Table 3:** Previously suggested pedigree symbols for TGD patients.

	Transgender man (assigned female at birth)	Transgender woman (assigned male at birth)	Non-binary or other gender-diverse identities (assigned female at birth)	Non-binary or other gender-diverse identities (assigned male at birth)
Bennett et al. [8]				
Comments	Recommend symbol representing “phenotypic gender”, which is ambiguous Assuming sex chromosomes may be inaccurate and cause clinician discomfort [9, 10]			
Provenzale et al. [11]				
Comments	Denoting sex assigned at birth in the centre could be interpreted as considering that this is core to the patient’s identity Symbols could easily be misdrawn/misinterpreted			
Barnes et al. [7]				
Comments	Use symbol that aligns to patient’s gender Annotation to indicate sex assigned at birth is preferable to sex chromosomes Participants indicated that diamond shape did not seem validating, as it is frequently used when gender is not known Recommendations supported by Bennett et al. [12]			
Tuite et al. [13]				
Comments	“Superscript symbols” eliminate the need for annotation Deviation from standardised pedigree and astrological symbols may cause confusion Triangle could be confused for “pregnancy not carried to term”			
von Vaupel-Klein & Walsh [1]				
Comments	Incorporate Barnes et al. [7] recommendations and suggest alternative for non-binary individuals with less potential for confusion and feeling of gender identity being invalidated			

The majority of TGD participants interviewed by Barnes et al. [7] preferred a single shape that reflects gender identity with annotation of sex assigned by birth. This both validates patient identity and provides information on sex assigned at birth, without assuming karyotype. This group reached consensus that trans men should be represented with a square, and trans women with a circle, and sex assigned at birth should be annotated beneath the symbol (as is standard for other relevant annotations).

Recent literature from the US suggest a diamond to represent non-binary patients or other gender-diverse identities [7, 12]. However, participants of Barnes et al. [7] expressed that a diamond did not seem validating, as it is used when gender is unknown, or not clinically relevant. This could also cause confusion for those interpreting the pedigree, leading to patients denoted as a diamond being omitted from risk assessment. Tuite et al. [13] suggest the alternative of an inverted triangle. However, this may be confused with the triangle which is standardly used to indicate a pregnancy not carried to term. Therefore, this group agreed that an additional symbol, beyond existing standards, should be used to denote non-binary patients or other gender-diverse identities.

Von Vaupel-Klein & Walsh [1] proposed a hexagon with annotation of sex assigned at birth as an easily recognisable and distinct pedigree a symbol. With the addition of a key/legend this could be an efficient and clear symbol to use for non-binary and gender-diverse patients. Moreover, a hexagon may be preferred by these patients, as it is a determinant of a gender identity, distinct from other symbols. There was some debate about the use of the hexagon due to the feasibility of drawing the symbol, and the potential of a poorly drawn hexagon to resemble a circle. Nonetheless, consensus was reached that a hexagon would be appropriate to use until further research is carried out. Further research involving TGD patients and members of the public, as well as clinicians, will be crucial to reach international consensus on the appropriate pedigree symbol.

There was strong consensus that annotations of “AFAB” or “AMAB” to indicate “assigned female at birth” or “assigned male at birth” should be used. These are preferrable to annotations regarding transition such as “FTM” or “MTF” to indicate “female to male”/“male to female”, or sex chromosomes. Given that around 1 in 400 newborns are affected by sex chromosome anomalies [9], making assumptions about sex chromosomes may be inaccurate. In addition, the acronyms FTM/MTF can invalidate the gender identity of TGD people, suggesting that their gender has “changed”, in contrast with the individuals’ reality that their gender identity has always been the same. TGD participants have expressed clearly that it is the responsibility of the genetic counsellors to explain the relevance of explicitly gathering sex assigned at birth [7]. There was strong consensus that it is important for patients to be advised that sex assigned at birth is recorded on pedigrees for accurate risk assessment and recommendations.

Concern was raised that pedigree drawing software may not accommodate the recommendations agreed by this group. It was agreed that, in such circumstances, clinicians should make reasonable efforts to manually alter electronically drawn pedigrees, recording in clinical notes where this is not possible. Furthermore, there was consensus that where pedigree drawing software cannot accommodate these recommendations, genetics services should encourage software developers to consider modifications.

### Clinical information

In a study of 21 TGD patients, self-reports from participants outlined their experiences and what they require from healthcare professionals (HCPs) to make a positive, safe, and respectful healthcare encounter [14]. Participants emphasised the importance of being accepted for who they are, which included HCPs using correct names, pronouns, and titles. There was consensus that clinicians should use the title, name, pronouns, and family relationship terms (i.e., brother/sister/sibling, mother/father/parent) that patients state their preference for, and these should be politely clarified if preference is not known. Some HCPs may feel discomfort in asking all patients their names, pronouns, and title. However, many cisgender patients use names that differ from their legal names and may feel discomfort at being referred to by an incorrect title. It was suggested that a question such as “is there anything I should know about how you like to be referred to?” can be a helpful alternative to gather this information. The consensus was also reached that name, gender, and title should be updated within clinical genetics records if requested by the patient. It was acknowledged that this may not always be possible due to restrictive clinical software, and it was therefore agreed that it should be documented if a request was made but not fulfilled.

Under The Gender Recognition Act (2004) it is an offence to disclose a person’s gender history when that information has been acquired in an official capacity, such as during the provision of healthcare [15]. Gender history can only be disclosed in healthcare settings when; it is to another HCP, for medical purposes, and there is a reasonable belief that the patient has consented to the disclosure. The consensus was reached that gender history should be treated with appropriate confidentiality, and clinicians should seek consent before recording, storing or sharing information about gender history.

Cancers of the breast, prostate, ovary, and endometrium are frequently a focus within cancer genetics. Clinicians should be mindful that discussions of organs that are incongruent with a patients’ gender identity should be handled with sensitivity to avoid exacerbating gender dysphoria [16]. Sensitivity and cultural competency is required to prevent negative experiences in clinical settings- a frequent issue faced by TGD patients, which reduces engagement with, and trust of, health services [14, 17, 18]. As with FHQs and pedigrees, there was consensus that it is the responsibility of clinicians to ensure that patients understand the relevance of clinical questions asked and examinations performed [1]. In addition, it was agreed that clinicians should be mindful not to ask for details of gender history where this is not relevant to care, and that questions about gender history should be asked clearly and directly, avoiding assumptions. Given the impact of gender-affirming treatment (GAT) on cancer risk, the relevance to family planning, and the potential for the intersection between gender care and clinical genetics [19], the consensus was reached that clinically relevant information can include details of GATs, gamete storage and care of gender identity specialists.

### Breast tissue management

A sparse, but growing body of research suggests that TGD people on gender-affirming hormone treatment (GAHT) have higher rates of breast cancer than cisgender men, but lower risks than cis women [20, 21], with younger ages of diagnosis observed [21]. There has previously been a lack of formal breast screening guidelines for TGD patients. Increasingly, literature recommends offering breast screening equivalent to cis women for TGD patients with breast tissue; including transmasculine patients who have not had chest surgery, and transfeminine patients who have breast tissue growth following GAHT [22–24]. Coad et al. [19] suggest offering TGD patients with breast tissue the same breast screening as cis women with an equivalent family history of relevant cancers. This group agreed that TGD patients assigned female at birth who have not had gender-affirming chest surgery should be offered the same breast screening as cis women with equivalent risk, as risk remains in the breast tissue. In addition, consensus was reached that TGD patients assigned male at birth who have breast tissue following 5 years of GAHT should be offered the same breast screening as cis women with equivalent inherited risk, due to the parity of breast development and risk after 5 years of exogenous oestrogen use [25].

Gender-affirming breast/chest surgery also has significant impacts on cancer risk. Consensus was *not* reached on whether TGD patients assigned female at birth who have had chest surgery should be offered the same screening as cis men. There was concern that whilst transmasculine chest surgery reduces breast cancer risk [23], the extent of risk reduction is not as great as risk-reducing mastectomy [26] as in most cases some tissue is retained to construct a masculine chest contour [27], and breast cancer in trans men after chest surgery is seen [21, 28]. However, the appropriate screening modality for these patients is unclear as mammography is difficult following masculinising chest surgery [22]. Attendees felt that to vote on best practice recommendations, more data was required regarding the average volume of tissue left after chest surgery, the impact of masculising hormone treatment on risk, and the efficacy of screening different methods in this cohort.

Breast screening for transmasculine patients is often incongruent with their gender identity, and may lead to increased gender dysphoria, and subsequently reduced adherence to screening recommendations [29]. Additionally, both transmasculine and transfeminine patients may be reluctant to pursue breast screening due to experienced or anticipated discrimination from HCPs [30]. Indeed, multiple studies have reported lower adherence to breast screening in the TGD population compared to the cisgender population [29, 31, 32]. Attendees acknowledged that there is great need for cancer screening services to adapt to meet the needs of trans patients. Whilst this is beyond the scope of this meeting, there was high-level consensus that TGD patients should be signposted to inclusive breast/chest awareness resources, such as the “Self Checkout” developed by CoppaFeel (available at https://self-checkout.coppafeel.org/onboarding-welcome).

TGD patients are often referred for genetic testing specifically to guide gender-affirming surgery (GAS) decisions, and decisions about surgery often play into their choices about genetic testing [33]. In addition, TGD patients are often seen at younger ages than typical for cancer genetics clinics as they were making decisions regarding GATs [33]. Genetics clinicians are well placed to contribute to these discussions and can refer for screening or risk-reducing surgeries. However, working in conjunction with gender identity specialists, endocrinologists, surgeons, radiologists, and with the patient may lead to better outcomes. For example, referring transmasculine patients for risk-reducing mastectomy has the benefit of reducing breast cancer risk, but would not result in a chest with a masculine appearance. Involving gender identity specialists and surgeons with expertise in GAS could also allow for the option of lipofilling for desired aesthetic outcome [19]. Similarly, whilst risk-reducing mastectomy and reconstruction is an option for transfeminine patients with GAHT-induced breast growth, timely intervention could allow for risk-reducing mastectomy and reconstruction prior to starting GAHT [19]. This approach could negate the cancer risks caused by GAHT and breast growth whilst reducing gender dysphoria by facilitating other feminising effects of GAHT and providing breast reconstruction. There was strong consensus that using a shared decision model involving the patient and relevant specialists is best practice for making breast/chest surgical management decisions. Additionally, attendees felt it appropriate to consider risk-reducing mastectomy at earlier ages than typical, if this is in alignment with the patient’s plans for gender-affirming care.

### Gynaecological and prostate management

In transmasculine people who have not had bilateral salpingo-oophorectomy or hysterectomy, there remains a risk of ovarian and endometrial cancer, respectively. There is contrasting data regarding the impact of testosterone on endometrial cancer risk. Despite a well-established association between higher testosterone levels in postmenopausal cis women and endometrial cancer [34, 35], there appears to be no evidence of increased incidence of endometrial cancer in trans men [36]. Similarly, there is currently no clear evidence to suggest higher rates of ovarian cancer in trans men [36], despite concerns that testosterone potentially drives ovarian carcinogenesis via endometrial epidermal growth factor receptors [37, 38]. However, measuring endometrial and ovarian cancer rates in TGD patients is difficult, as many opt for oophorectomy within the first few years of GAHT [39], and may also opt for hysterectomy. In addition, much of the data has come from registry studies that lack detail of sex assigned at birth and previous gynaecological surgeries [36].

Current evidence does not support routine ovarian cancer screening for cisgender or TGD people [40–42]. Additionally, within the NHS there is no routine endometrial cancer screening in cisgender women, and guidelines for endometrial thickness monitoring for TGD people vary [43–45]. However, hysterectomy and/or bilateral salpingo-oophorectomy (BSO) is offered to cisgender women with a high risk of endometrial and/or ovarian cancer [46, 47]. These surgeries are generally offered from age 35 at the earliest [46–48], to balance the risks of cancer against the implications for childbearing and surgical menopause. There was consensus that it may be appropriate to consider risk-reducing gynaecological surgeries for TGD patients at earlier ages than with cis women, given that hysterectomy and BSO may already be part of someone’s plan for transition.

As the removal of the prostate is not recommended as part of transfeminine GAS, it is important to consider risks in TGD patients with prostate. Data from a Dutch cohort reported a fivefold lower risk of prostate cancer in trans women on GAHTs compared to cis men [49]. However, reported cases of prostate cancers in trans women are often aggressive and detected late, resulting in high rates of mortality [50, 51]. Nonetheless, the World Professional Association of Transgender Health (WPATH) and the Endocrine Society advocate for all TGD persons with a prostate to be offered the same screening as cis men [39, 52]. For those with a genetic predisposition to prostate cancer, this is particularly relevant. Cis men with an inherited high risk of prostate cancer are offered prostate specific antigen (PSA) testing [53]. However, concerns have been expressed regarding the efficacy of PSA testing in TGD patients taking exogenous hormones, as the reduction in testosterone levels appears to lower PSA levels [54]. Digital rectal examination (DRE) is also of limited use in TGD patients due to prostate atrophy following GAHT and/or genital reconstructive surgery [54]. Given the complexities of interpreting PSA levels in TGD patients taking GAHTs, and the limited utility of DRE, there was strong consensus that it is best practice for TGD patients with a high risk of prostate cancer to be referred to a specialist to discuss options for prostate screening.

### Patient pathways

Medical GATs can greatly improve TGD patients’ well-being, and therefore denying access to such treatments based on unsubstantiated data can be harmful and unethical [20, 55]. Patients of any age should not feel pressured to have genetic testing in order to access GATs, and it is unethical for genetic testing to pose an additional barrier to accessing treatment [19]. There was a strong consensus that inherited cancer predisposition should not be a barrier to accessing GAT. In addition, given the regrettably long waitlists for NHS gender and genetics care, attendees felt it is appropriate for genetics clinicians to consider treating cases with priority if genetics input is delaying GAT. Similarly, attendees agreed that it is appropriate for genetics clinicians to request that gender identity services treat cases with priority if gender care is delaying timely cancer risk reduction. As GATs and inherited cancer risk management options can intersect (particularly in the context of breast, prostate, womb, and ovarian cancer), there was consensus that it is appropriate for patients’ gender care teams to be consulted (with patient consent) whilst they are under the care of clinical genetics. At a minimum, this should be copying relevant gender clinicians into correspondence, where consent is provided. However, cases may require direct contact and/or discussion, depending on complexity and urgency.

Data on cancer incidence for TGD people with a genetic predisposition are particularly sparse [56], leading to challenges for genetics services managing the care of TGD patients with a family history of cancer. TGD patients report disappointment and frustration with the lack of information available about their cancer risks, and its implications for making informed decisions on risk management and GAT [57]. Similarly, genetic counsellors have expressed discomfort at making risk management recommendations based on the limited information available [33]. Further research is imperative to accurately establish the cancer risks for TGD patients, particularly in the context of familial risk. To make informed decisions, patients should be given all current relevant information about the risks and benefits of GATs and cancer risk management [33, 58]. Genetic counsellors are well placed to contribute to these discussions, but a multidisciplinary team (MDT) approach is optimal to ensure input from a range of clinicians with specialist expertise [19]. There was strong desire from attendees for an MDT meeting involving relevant specialists, with the majority of the group expressing preference for a regularly scheduled national MDT meeting. It was acknowledged that such a meeting would require adequate funding and resources.

### Education

A significant barrier to healthcare for TGD patients is limited clinician knowledge of, and sensitivity to, their specific healthcare needs [59, 60]. The 2015 US Transgender Survey revealed that 24% of respondents had to teach an HCP about their specific healthcare needs in order to receive appropriate care [61]. Much of the literature focussing on HCPs knowledge and education regarding TGD healthcare has done so under the wider umbrella of LGBTQ+ education. Only 8% of UK oncologists reported that they felt confident in their knowledge of the specific healthcare needs to LGBTQ+ cancer patients [62]. This lack of knowledge seems to stem from inadequate inclusion of LGBTQ+ issues within healthcare training [63]. Genetic counsellors have reported limited training about TGD healthcare [33, 64–66]. Cancer genetic counsellors in particular felt underprepared due to a lack of training on TGD healthcare and insufficient data regarding the impact of GATs on risk, especially in the context of assessing the cancer risk associated with a pathogenic variant [33, 64–67].

Exclusion of LGBTQ+ and TGD-specific topics from healthcare curricula may contribute in part to the healthcare inequalities faced by TGD populations. Inclusion of TGD topics within curricula not only improves HCP knowledge, skills, and attitudes, but can also empower HCPs to address TGD healthcare inequalities and barriers to care [68]. Online modules developed specifically for the education of genetic counsellors regarding gender-affirming care have proven effective at improving knowledge and self-efficacy [69]. TGD-specific training within medical and genetic counselling curricula and continuing professional development (CPD) may therefore provide great benefits to patients and clinicians alike. There was a particularly strong desire from this group for relevant education via CPD, but also a considerable desire for education within genetic counselling and medical training. It was acknowledged that administrative staff may also benefit from such training, to ensure all interactions within healthcare settings are positive, respectful and appropriate.

### Strengths, limitations and future directions

These are the first UK consensus guidelines for the management of TGD patients with inherited cancer risks. It is hoped that these recommendations will help to address previous inconsistencies in practice and improve equity and access to care for TGD patients. This guidance is grounded in existing literature, service evaluation, and the diversity of the meeting attendees, including relevant clinical specialists, patient advocates, and members of the TGD community. A greater proportion of TGD individuals present would have been preferred for a greater representation of lived experience. Nonetheless, the diversity of experience, perspective, and expertise present fuelled constructive discussion and informed voting, which led to consensus being reached on the majority of statements. We hope that work such as this will improve trust between members of the TGD community, clinical genetics services, and researchers, facilitating greater community outreach and participation in the future.

The aim of this meeting was to develop guidelines for adults with inherited cancer risk. However, it was recognised that many of the consensus views are also applicable to children, and to other aspects of clinical genetics beyond cancer. The final statements were therefore reviewed and received endorsement from the Clinical Genetics Society (CGS) Council and the Association of Genetic Nurses and Counsellors (AGNC) Committee.

A major limitation of the guidance is the scarcity of data on which to base discussions and recommendations. There is a stark need for further research, particularly on the impact of GAT on cancer risks for those with an inherited predisposition, and into the experiences of TGD patients accessing cancer genetics services. Given these limitations, it is of particular importance that these recommendations are revisited as further evidence emerges.

It was recognised that there is a great need (and desire) for training regarding TGD-specific healthcare issues and cultural competency. Including these topics within genetic counselling training programmes and medical curricula is an important long-term goal. In the meantime, relevant topics should be included within CPD to improve the awareness, cultural competence, and knowledge of our existing workforce. In addition, it was recognised that there is sufficient demand and interest for a national MDT of relevant specialists to discuss the management of TGD patients with inherited cancer risk. Acquisition of funding and resources is imperative to take this further step towards high standards of care for TGD patients within cancer genetics.

## Supplementary information

Supplementary

## Data Availability

All data are included in this published article.
