# Supplementary material for: UK recommendations for the management of transgender and gender-diverse patients with inherited cancer risks
Source: BJC Rep. 2023 Jun 22;1:1. doi: 10.1038/s44276-023-00002-0 (PMC11523990; doi:10.1038/s44276-023-00002-0)
Supplement: Supplementary file 1 — Supplementary [file 44276_2023_2_MOESM1_ESM.docx]

*“The sex someone is assigned at birth does not always match their gender identity. Both are important to us when we make cancer family history assessments. When we look at a family history of cancer, we look for clues of possible inherited causes. Some clues can vary depending on a person’s sex assigned at birth. For example, it is rarer to see breast cancer in someone who’s sex assigned at birth is male. This means it is more likely that their cancer was caused by a genetic factor, than if their sex assigned at birth was female.”*

Example of a statement explaining why sex assigned at birth and gender identity are asked on a family history questionnaire - adapted from St George's Family History Questionnaire Service, available at <https://fhqs.org/faq>
